# Adaptive Geometric Tessellation for 3D Reconstruction of Anisotropically Developing Cells in Multilayer Tissues from Sparse Volumetric Microscopy Images

**DOI:** 10.1371/journal.pone.0067202

**Published:** 2013-08-05

**Authors:** Anirban Chakraborty, Mariano M. Perales, G. Venugopala Reddy, Amit K. Roy-Chowdhury

**Affiliations:** 1 Electrical Engineering, University of California, Riverside, California, United States of America; 2 Botany and Plant Sciences, University of California, Riverside, California, United States of America; Centrum Wiskunde & Informatica (CWI) & Netherlands Institute for Systems Biology, Netherlands

## Abstract

The need for quantification of cell growth patterns in a multilayer, multi-cellular tissue necessitates the development of a 3D reconstruction technique that can estimate 3D shapes and sizes of individual cells from Confocal Microscopy (CLSM) image slices. However, the current methods of 3D reconstruction using CLSM imaging require large number of image slices per cell. But, in case of *Live Cell Imaging* of an actively developing tissue, large depth resolution is not feasible in order to avoid damage to cells from prolonged exposure to laser radiation. In the present work, we have proposed an anisotropic Voronoi tessellation based 3D reconstruction framework for a tightly packed multilayer tissue with extreme z-sparsity (2–4 slices/cell) and wide range of cell shapes and sizes. The proposed method, named as the ‘Adaptive Quadratic Voronoi Tessellation’ (AQVT), is capable of handling both the sparsity problem and the non-uniformity in cell shapes by estimating the tessellation parameters for each cell from the sparse data-points on its boundaries. We have tested the proposed 3D reconstruction method on time-lapse CLSM image stacks of the Arabidopsis Shoot Apical Meristem (SAM) and have shown that the AQVT based reconstruction method can correctly estimate the 3D shapes of a large number of SAM cells.

## Introduction

The causal relationship between cell growth patterns and gene expression dynamics has been a major topic of interest in developmental biology. However, most of the studies in this domain have attempted to describe the interrelation between the gene regulatory network and cell growth and deformation qualitatively. A proper quantitative analysis of the cell growth patterns in both the plant and the animal tissues has remained mostly elusive so far. Information such as rates and patterns of cell expansion play a critical role in explaining cell growth and deformation dynamics and thereby can be extremely useful in understanding morphogenesis. The need for quantifying these biological parameters (such as cell volume, cell growth rate, cell shape, mean time between cell divisions etc.) and observing their time evolution is, therefore, of utmost importance to biologists.

For complex multi layered, multi cellular plant and animal tissues, the most popular method to capture individual cell structures and to estimate the aforementioned parameters for growing cells is the Confocal Microscopy based *Live Cell Imaging*. Confocal Laser Scanning Microscopy (CLSM) enables us to visually inspect the inner parts of the multilayered tissues. Through this technique we can image tissues as a collection of serial optical slices (also known as the ‘Z-Stack’), which can then be used for analysis. Live cell imaging is a class of microscopy, where the same living cells are observed and imaged at regular time intervals over several hours to monitor their motion or displacement and to visualize cell growth and division dynamics.

Recently, there has been a substantial amount of work in automated processing and analysis of cellular images – though mostly on image segmentation and cell tracking. Methods such as [1], [2] show that individual cells can be efficiently segmented in a multicellular field and [3], [4] provide automated methods to track individual cells in time. Estimation of cell shape and volumes as a function of time is most fundamental to understanding of the growth process. Due to the large quantity of data collected during the growth of a tissue, computational methods for robust estimation of 3D cell structures and cell volumes are absolutely necessary in order to obtain statistically significant results of these growth parameters.

Inspite of the extreme usefulness of CLSM based live cell imaging for analysing such tissue structures, there are number of technical challenges associated with this imaging technique that makes the problem of cell shape estimation non-trivial. To keep the cells alive and growing, we have to limit the laser radiation exposure to the specimen, i.e. if dense samples in one time point are collected, it is highly unlikely that we will be able to get time lapse images as the specimen will not continue to grow in time due to high radiation exposure. Therefore the number of slices in which a cell is imaged is often very low (2–4 slices per cell). Again, the fluorescent signal fades as we image the deeper layers of the tissue, thereby bringing in the problem of very low SNR in parts of the confocal image stack. Please note that in some cases, a two-photon excitation microscopy or light sheet microscopy can be better choices for live-cell imaging for more efficient light detection and less photo-bleaching effect. But, a large number of data sets exist that are imaged using CLSM or exhibit the characteristic of our data and our method can be useful in analyzing them. We have found that two photon excitation is toxic to SAM cells than the single photon CLSM and since the SAM is surrounded by several developing flower buds, the side ward excitation may not be possible. Also, by designing an image-analysis method that is capable of handling the worse quality data, we can ensure that same or better accuracy can be achieved on a data-set having superior image quality and resolution. Thus, from an image analysis perspective, we are looking at a very challenging problem where we want to obtain a 3D surface reconstruction of arbitrary cell shapes from a set of very sparsely sampled data points in presence of unavoidable imaging noise. Also, the reconstruction pipeline must be fully automated. In most cases, manual analysis (which has been the trend) is usually extremely tedious and, often, only provides qualitative trends in the data rather than precise quantitative models.

In this study, we have looked at the problem of 3D reconstruction of a tightly packed multi-layer tissue from its Z-sparse confocal image slices. As a special example, in this paper we have proposed a novel, fully automated cell resolution 3D reconstruction framework for Shoot Apical Meristem (SAM) of Arabidopsis Thaliana. SAM, also referred to as the stem cell niche, is a very important part of a plant body plan because it supplies cells for all the above ground plant parts such as leaves, branches and stem. A typical Arabidopsis SAM is a densely packed multi layered cell cluster consisting of about five hundred cells where the cell layers are clonally distinct from one another. The tight tessellation of cells in SAM enabled us to estimate the 3D structure of individual cells using the slice information of the cell as well as that of its nearest neighbors. The 3D estimation is based on prior geometrical tessellation models, the parameters for which are estimated from the sparse image data to hand and then this model is used to partition the 3D SAM structure into individual cellular regions.

Being motivated by the methods in [5], [6], we first assume a ‘Voronoi’ tessellation model based on Euclidean distance metric to segment/reconstruct the 3D cell shapes. Through the results obtained on 3D sparse confocal stacks of SAM images we show that this model yields a good approximation of cell shapes where the shapes and sizes of the cells are uniform along all three axes of the cells and the major axes of growth of the neighbouring cells are isotropic. But, in practice, this is not always the case and the cells can have very anisotropic shape and growth, even in a close neighborhood. In such cases, the Voronoi tessellation using the Euclidean distance fails to generate accurate enough cell walls.

We, therefore, propose an anisotropic Voronoi Tessellation defined on a quadratic distance metric to capture the growth anisotropy of individual cells along all of their axes. We show that the parameters of this metric can be estimated from the sparse set of confocal image slices of the individual cells and the tessellation based on this metric can provide very accurate 3D cell shapes as quadratic surfaces even in the case of non-uniform cell shapes, sizes or growth along different cell axes. The tessellation, named as the ‘Adaptive Quadratic Voronoi Tessellation’ (AQVT) and the 3D reconstruction technique based on it, presented in this paper, provide accurate enough 3D reconstructed cell shapes and sizes in the SAM as validated through the experiments in Section 5.3. We also show that the proposed anisotropic Voronoi tessellation (AQVT) can also be applied on tissues, where the cell shapes in the tissue follow a standard Euclidean distance based Voronoi tessellation.

While this method is motivated by our previous work in [7], there are fundamental differences in both theory and implementation between [7] and the present work based on AQVT. The main differences are as follows. 1. The AQVT poses and solves the problem of 3D segmentation/reconstruction in the standard framework of geometric tessellation, which is very intuitive for such type of problems and has solid theoretical basis in the literature. 2. The method described in [7] is an iterative method that can have long runtime depending on desired level of accuracy and chosen parameters (e.g. step-size in the deformation stage). AQVT based reconstruction method described in this paper is a single-step process and hence has lesser execution time in comparison to [7]. 3. While there are a number of user defined parameters and thresholds required for the method in [7], AQVT does not require any user input other than the sparsely sampled segmented cell slices for reconstruction, thereby making the present method less ambiguous and easy to use. It can be shown that under a very specific criterion, [7] can generate similar results as the proposed method, which is discussed in detail in Section 4.2.3.

## Problem Formulation

### 2.1 Goal

The objective of the present work is to obtain a fully automated cell-resolution 3D reconstruction/segmentation of a tightly packed anisotropically growing tissue from initial 2D segmentations and slice to slice correspondences of its sparsely Z-sampled confocal image slices.

### 2.2 Research Challenges and Contributions

**Figure 1:**
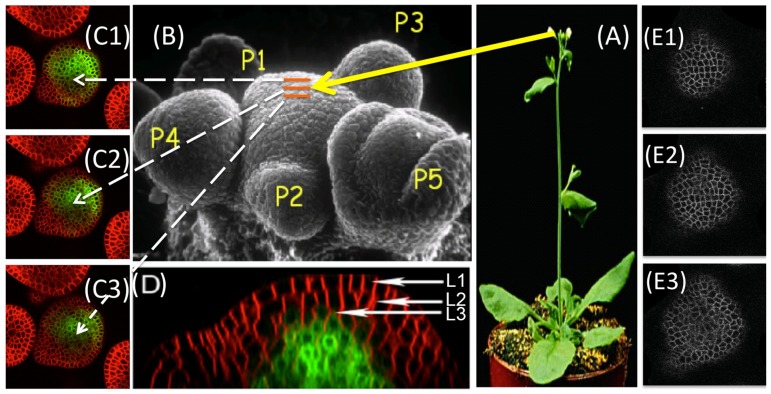
Shoot Apical Meristem (SAM): A multilayer cell cluster. (A) SAM located at the top of the shoot of Arabidopsis, (B) A detailed surface view showing different regions of SAM, (C1–C3) Three consecutive slices of SAM, each 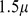
m apart, obtained through CLSM technique, (D) A cross sectional side view of SAM, which clearly shows the multiple layers (L1, L2, L3) of tightly packed stem cells and their shapes, (E1–E3) The visible cell walls of individual cells in 3 sparsely sampled consecutive slices of the SAM obtained from the 3D CLSM live imaging dataset.

There are several methods of shape and size estimations for individual cells such as impedance method [8] and light microscopy methods [9]. Methods such as [10] are used to study changes in cell sizes in cell monolayers. In live plant tissues, a number of work focussed on the surface reconstruction [11], [12]. But we are looking at a much more challenging problem where the subject of study is a dense cluster of cells. Plant meristem is one example of such cell clusters where hundreds of small cells are densely packed into a multilayer structure (Figure 1). In such cases, now-a-days, the most popular practice is to use Confocal Laser Scanning Microscopy (CLSM) to image cell or nucleus slices at a very high spatial resolution and then reconstruct the 3D volume of the cells from those serial optical slices which has been shown to be reasonably accurate [13]–[15].

However, performance of the current imaging based 3D reconstruction techniques depends heavily on the availability of a large number of very thin optical slices of a cell and the performance rapidly deteriorates in the cases where the number of cell slices becomes limited. This problem is very common especially in CLSM based *live cell imaging* when the time gap between successive observations is small. In order to keep the cells alive and growing for a longer period of time and obtain frequent observations, a cell cannot be imaged in more than 2–4 slices, i.e., high depth-resolution and time-resolution cannot be achieved simultaneously.

A very recent method [14] accurately reconstructs the Shoot Apical Meristem of Arabidopsis. This method uses a dataset containing fine slice images acquired from 3 different angles, each at a Z-resolution of 1 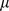
m. They have reported 24 hours as the time resolution in imaging. But, for analyzing the growth dynamics of cell clusters where the time gap between successive cell divisions is in the range of 30 to 36 hours, we need a much higher time resolution in imaging in order to capture the exact growth dynamics. To obtain longer cell lineages at high time resolution we may have to sacrifice the spatial or depth resolution and hence the number of image slices in which a cell is present can be really small. With such a limited amount of image data, the existing 3-D reconstruction/segmentation techniques cannot yield a good estimate of cell shape. In the present work we have addressed this problem of reconstructing plant cells in a tissue when the number of image slices per cell is very limited.

There is a basic difference between the segmentation problem at hand and a classical 3D segmentation scheme. A classical method solves the segmentation problem using the pixel intensities and cannot work when no intensity information is provided for a majority of 3D pixels in the image. In such situations, the most intuitive way to perform the segmentation is to first segment sections in the image with known intensity information using a classical segmentation scheme, and then to extrapolate between these sparse segments using a known geometric model or a function which could be generic or data-specific. In this work, we have shown that a quadratic Voronoi tessellation is a very accurate choice for such a geometric model for segmenting a tissue with anisotropically growing tightly packed cells starting with a sparse set of 2D Watershed segmented slices per cell.

The Shoot Apical Meristem is a multilayer, multicellular structure where the cells are tightly packed together with hardly any void in between. Motivated by this physical structure of SAM, we propose our novel cell resolution 3D reconstruction in a geometric tessellation framework. A tessellation is a partition of a space into closed geometric regions with no overlap or gap among these regions. In case of the SAM tissue, each cell is represented by such a closed region and any point in the 3D SAM structure must be the part of one and only one cell. In fact, there are some recent works in the literature as [5] which predicted that the 3D structures of Arabidopsis SAM cells could be represented by convex polyhedrons forming a 3D ‘Voronoi’ tessellation pattern.

A Voronoi tessellation is one of the simplest form of partitioning of the metric space, where the boundaries between two adjacent partitions are equidistant from a point inside each of these regions, also known as the ‘sites’. In [5], [6], these sites are the approximate locations of the center of the cell nuclei about which the tissue is tessellated into individual cells. However, this work used a dataset where both the plasma membrane as well as the nucleus of each cell is marked with fluorescent protein, whereas, in our case, only the plasma membrane is visible under the confocal microscope.

In this work, we present and evaluate a fully automated cell resolution 3D reconstruction framework for reconstructing the Arabidopsis SAM where the number of confocal image slices per cell is very limited. The framework comprises of different modules such as cell segmentation, spatial cell tracking, SAM surface reconstruction and finally a 3D tessellation module. We evaluate the Euclidean distance based Voronoi tessellation model on our dataset and then from its limitation, we continue to propose a quadratic distance based anisotropic Voronoi tessellation, where the distance metric for each cell is estimated from the segmented and tracked sparse data-points for the cell. This method is applicable to the densely packed multi-cellular tissues and can be used to reconstruct tissues without voids between cells with sufficiently high accuracy. Note that, for the proposed 3D reconstruction module, we start with a handful of data-points on each segmented cell which are pre-clustered through the cell tracking method (i.e. an incomplete segmentation of the 3D) whereas, the final output of our algorithm is a complete tessellation of the entire 3D structure of the SAM, where each cell is represented by a very dense point cloud. These point clouds for individual cells can be visualized by 3D convex polyhedrons that approximate the shape of the cells.

### 2.3 Organization

The rest of the paper is organized as follows. Sections 3.1 and 3.2 describe the overview of our approach and challenges associated with various steps in it. The mathematical and algorithmic details of the proposed reconstruction method are provided in Sections 4.1 and 4.2. Finally, we present the experimental results and validation of our approach followed by a concluding discussion.

## Overview of the Proposed Method

To properly understand the research challenges and our contributions, we first explain the data (Section 3.1). We also briefly describe the necessary preprocessing stages such as cell segmentation and cell tracking that generate the final data structure as an input to the 3D reconstruction pipeline.

### 3.1 Imaging Setup and Preprocessing

The SAM of *Arabidopsis Thaliana* consists of approximately 500 cells and they are organized into multiple cell layers that are clonally distinct from one another. By changing the depth of the focal plane, CLSM can provide in-focus images from various depths of the specimen. To make the cells visible under laser, fluorescent dyes are used. The set of images, thus obtained at each time point, constitute a 3-D stack, also known as the ‘Z-stack’. Each Z-stack is imaged at a certain time interval (e.g. 3 hours between successive observations) and it is comprised of a series of optical cross sections of SAMs that are separated by approx. 1.5 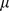
m (Figure 1). A standard shoot apical meristematic cell has a diameter of about 5 – 6 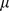
m and hence in most cases, a single cell is not visible in more than 3–4 slices when the tissue is sparsely imaged at 1.5 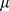
m to avoid photodynamic damage to the cells. To account for any minor shift in the alignment of the images in the 3-D stack, each stack is registered by a method of maximization of mutual information [16].

As we are interested in computing volume of every cell in the SAM cell cluster, we need to segment out all the cells in each slice. We can employ various segmentation algorithms like Watershed [17], Level-Set segmentation [1] etc. which have their own advantages and disadvantages. Although the method we propose here is independent of the segmentation strategy we choose to employ, we have preferred Watershed [18] over level-set segmentation as it produces more accurate and realistic cell boundaries for our SAM confocal data. Please note that, the contribution of our method lies in the post segmentation and tracking stage though a better segmented data is guaranteed to improve the performance of both the tracking and 3D reconstruction methods.

In order to find a cell's correspondence across multiple slices in both the spatial and temporal direction, we have used our local-graph matching based robust cell tracking algorithm [4], [19]. This algorithm starts by finding out a *seed cell pair* between two SAM slice images using ‘local graph matching’ and progressively moves outward from the seed-pair to obtain correspondences between neighboring cells until all the cells are tracked. This method is robust because it fuses tracking results over the entire 4-D image stack and thereby minimizes the chances of losing a cell in any of the slices caused by poor segmentation of noisy data. Another advantage comes from the batch processing capability of this method which enables us to reconstruct a large number of SAM cells at a time.

### 3.2 Proposed 3D Reconstruction – Overview

Once the sparse image slices are segmented and tracked to generate the initial clustering of individual cell slices, the objective is to obtain full 3D reconstructions of these cells. As explained above, for live imaging with frequent observations in time, the number of slices in which a particular cell can be present is very small (e.g. 2–4 slices/cell). Unfortunately, the existing 3-D reconstruction methods are not capable of handling such sparsity in data. Motivated by the physical structures of the cells, we handle this issue by assuming a prior geometrical 3D tessellation model for the tissue, the parameters of which is estimated to fit the given sparse set of segmented cross sectional images.

In [5], [6], the authors have used a standard Voronoi tessellation technique to estimate the cell boundaries from the known information of the nucleus location for individual cells. Motivated by their work, we first show how a Voronoi tessellation can be fitted to our CLSM dataset which only have the partial cell wall information (Section 4.1.1, Section 4.1.2). Unlike the dataset used in [5], [6], we do not have the cell nuclei marked in our dataset. The standard affine Voronoi tessellation is not always accurate enough to reconstruct the cells in SAM as different cells in the tissue can have very diverse sizes as well as the neighbouring cells might not have isotropic growth directions. This motivates us to propose an anisotropic Voronoi tessellation model (Section 4.2) for the tissue, which is also a generalization of the standard Affine Voronoi tessellation. We show how to estimate the parameters (Section 4.2.1) of an anisotropic or quadratic distance function for individual cells from the sparse data-points on the boundaries of these cells. The proposed quadratic Voronoi tessellation approach, termed as the ‘Adaptive Quadratic Voronoi Tessellation’ (AQVT) is then used to cluster a dense point cloud obtained from within the estimated 3D surface of the SAM and thereby, to generate the final 3D shapes of each individual cell (Section 4.2.2).

## Detailed Methods: The 3D Reconstruction Framework

### 4.1 Voronoi Tessellation Based 3D Reconstruction

#### 4.1.1 A Brief Overview of Voronoi Tessellation and Its Properties

‘Voronoi Diagram’ is a geometric minimization diagram that splits its embedding space into different non-overlapping regions. Each of these regions is characterized by a generating point or an object also known as the ‘site’. All other points in each of these regions are closer to the site in its region than to any other site in the entire embedding space. The closeness of the points to the sites is computed using a distance metric. There can be different types of sites ranging from a point, a line to any complex geometric shape. Depending on the type of sites, the distance metric or the embedding space, several different variations of the Voronoi diagram can be defined. Detailed discussions on many of such variants can be found in [20]–[22].

Based on the characteristic of site and distance function, the locus of the points equidistant from two neighboring sites (also termed as the ‘bisectors’ or ‘edges’) can be hyperplanes or higher order hypersurfaces. We call the Voronoi diagrams with hyperplane bisectors as the ‘Affine Voronoi’ diagrams. The most common example of such an affine diagram is the Voronoi diagram of points based on the Euclidean distance metric.

Let there be 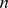 point sites in a space 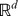, and the set of all sites 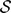 be 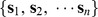. The Voronoi regions associated with these sites are represented as 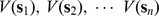 where,
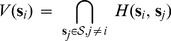
(1)where 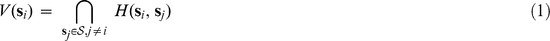 is the half-plane defined by,



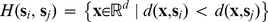
(2)The distance 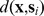 for a standard Voronoi diagram is the Euclidean distance defined by
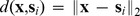



Again, the set of all points on the bisector between two Voronoi regions 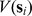 and 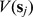 is given by,
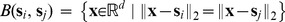
(3)


Some of the properties of the Euclidean distance based Voronoi diagram with point sites are as follows:

1. The line joining any two Voronoi sites 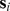 and 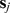 is always perpendicular to the Voronoi bisector/edge 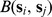,
2. The perpendicular distances from 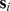 and 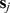 to 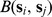 are equal to one another,
3. Any point 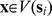 would satisfy

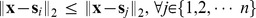
and
4. The Voronoi regions, thus produced, are convex polyhedrons and can be expressed as intersection of a finite number of open or closed half spaces.

**Figure 2:**
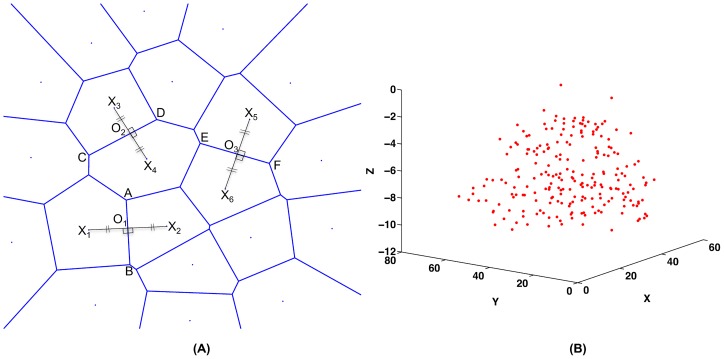
A schematic of Voronoi Tessellation and Estimated SAM cell centroids as Voronoi sites. (A) A Voronoi diagram based on the Euclidean distance metric for twenty one sites in 2D. The figures also show that the Voronoi edges are perpendicular to the line joining any two neighbouring sites. 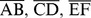 are three of the Voronoi edges and they are the perpendicular bisectors of 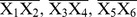 respectively. (B) Centroids are estimated for around two hundred cells in a SAM tissue, which are also the sites of Euclidean distance based Voronoi tessellation.

In Figure 2(A), we have shown some of the properties for a 2D Voronoi tessellation with twenty one sites (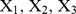 etc.). It can be observed that each of these Voronoi regions is a convex polygon.

#### 4.1.2 Voronoi Sites Estimation From Sparse Data

After segmentation and tracking, we are given a sparse set of 3D data-points that lie on the boundary between the neighbouring cells. Our objective is to fit a Voronoi tessellation to these data-points to obtain complete structures of individual cells, represented as Voronoi polyhedrons in 3D. Therefore, ideally the sparse data-points should lie on the bisectors between the neighbouring Voronoi regions. Given a very sparse set of data-points on the bisectors as in the case of ‘Live cell imaging’, the only way to reconstruct the Voronoi diagram is to first estimate the approximate locations of Voronoi sites, from which the Voronoi edges/bisectors can then be computed.

Given a set of generating sites, the construction of the Voronoi diagram can be done through several methods, the most popular of which being Fortune's ‘sweep line’ algorithm. The inverse problem, i.e. to obtain the locations of the sites given the Voronoi bisector, is, however, less studied in the literature. In [23], Evans et al. proposed a linear least-square technique to estimate the Voronoi sites by fitting a Voronoi diagram over a given tessellation pattern. Again, in [24], a number of algorithms have been proposed to obtain the Voronoi sites given the vertices of the Voronoi polygons. But neither of these methods is applicable to the sparse data that we have. These methods require the knowledge of the complete structures of the Voronoi polyhedrons, which is precisely the output that we are after. In fact, because of the extreme sparsity in our dataset, it is rather impossible to obtain unique estimates of the Voronoi sites for individual 3D cells.

In this situation, the knowledge about the physical structure of the SAM stem cells help us in obtaining approximate locations of the Voronoi sites, where each cell is represented as a Voronoi region. In [6], the authors observed that the SAM cells can be represented as Voronoi regions where the sites are located at the approximate centers of the cell nuclei. This observation, along with the fact that the nucleus, located in the central region of the cell, contributes to the majority of the size of a SAM cell motivates us to devise a simple strategy to estimate the approximate site locations, even when the nuclei are not imaged in the confocal image stack.

Given that the sparse set of points on the segmented and tracked slices of a cell 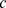 are 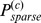 (the 3D data-point set 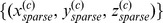), the approximate centroid location of the cell would be 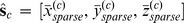, where the elements are the arithmetic means of 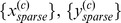 and 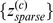 respectively. Thus, 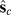 is also the estimated approximate location of the site corresponding to the Voronoi region representing the cell 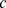. The centroids for approximately 200 cells from a SAM tissue is shown in Figure 2(B).

Now, the next step would be to generate a dense point cloud sampled from within the SAM structure and to cluster this dense set of 3D data-points into different Voronoi regions (representing individual cells) based on the estimated locations of the sites 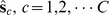.

#### 4.1.3 Generation of Dense Point Cloud To Be Partitioned Into Cells: Global Shape of SAM

**Figure 3:**
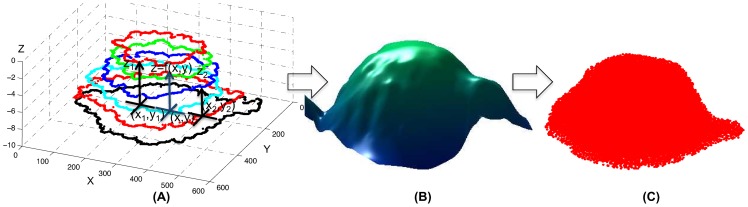
Generation of the dense point cloud from within the reconstructed SAM surface. (A) The SAM contours extracted from the confocal image stack using Level-Set segmentation, (B) The SAM surface is reconstructed using linear interpolation on a local neighbourhood of points on the SAM contours, (C) A very dense point cloud is extracted from within the reconstructed SAM surface which is clustered using the proposed reconstruction technique into individual cells.

At this stage, we estimate the 3D structure of the SAM by fitting a smooth surface to its segmented contours. The surface fitting is done in two steps. In step one, the SAM boundary in every image slice is extracted using the ‘Level Set’ method (Figure 3(A)). A level set is a collection of points over which a function takes on a constant value. We initialize a level set at the boundary of the image slice for each SAM cross section, which behaves like an active contour and gradually shrinks towards the boundary of the SAM. Let the set of points on the segmented SAM contours be 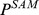 (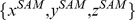).

In the second step, we fit a surface on the segmented points 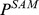. Assuming that the surface can be represented in the form 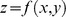 (where the function 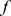 is unknown), our objective is to predict 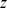 at every point 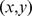 on a densely sampled rectangular grid of points bounded by 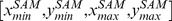. As the segmented set of data points are extremely sparse, this prediction is done using a linear interpolation on a local set of points on the grid around the point 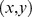. As the value (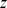) for the point 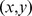 is approximated by a linear combination of the values at a few neighboring points on the grid, the interpolation problem can be posed as a linear least-square estimation problem. We also impose a smoothness constraint in this estimation by forcing the first partial derivatives of the surface evaluated at neighboring points to be as close as possible. A MATLAB visualization [25] of the surface is shown in Figure 3(B).

Once the SAM surface (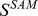) is constructed, we uniformly sample a dense set of 3D points (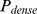, a visualization can be found in Figure 3(C)) such that every point in 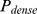 must lie inside 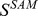. Thus, 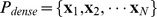 and the required output from the proposed algorithm is a clustering of these dense data points into 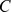 cells/clusters such that 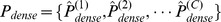 starting from the sparse set of segmented and tracked points 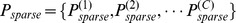 obtained from the confocal slice images of individual cells.

#### 4.1.4 Segmentation of The Dense Point Cloud Into Voronoi Cells

In this step, we partition the dense point cloud 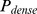, obtained in the last step, into 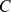 clusters based on the site locations 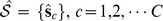, estimated in Section 4.1.2.

The 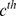 cell can be represented as a collection of dense data-points belonging to the 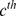 Voronoi region as
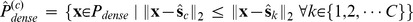
(4)


Once the dense point cloud belonging to each Voronoi region is obtained, we can construct convex polyhedrons with each of these dense point clusters (
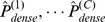
) to obtain the cell resolution 3D reconstruction of SAM.

### 4.2 An Adaptive Quadratic Voronoi Tessellation (AQVT) For Non-uniform Cell Sizes And Cell Growth Anisotropy

In a tissue like SAM, cells do not grow uniformly along all three axes (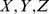). In fact, most of the cells show a specific direction of growth. Again, neighboring cells in SAM, especially in the central region (CZ), are not likely to grow along the same direction. Thus, even if a tessellation is initially an affine Voronoi diagram, it is not likely to remain so after a few stages of growth. Such cases of non-uniform cell sizes and anisotropic growth can be captured in a more generalized non-affine Voronoi tessellation called the ‘Anisotropic Voronoi Diagrams’. In the most general form of such diagram for point sites, the distance metric has a quadratic form with an additive weight [22].

Following similar notations used in previous sections, for a set of anisotropic sites 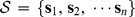 in 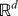, the anisotropic Voronoi region for a site 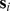 is given by,
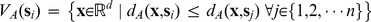
(5)where
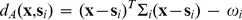
(6)

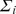
 is a 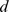 x 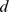 positive definite symmetric matrix associated with the site 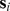 and 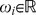. Thus each of the anisotropic Voronoi regions is parameterized by the triplet 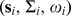. Further assuming 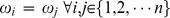, the distance function becomes



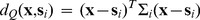
(7)As the bisectors of such a Voronoi diagram are quadratic hypersurfaces, these diagrams are called ‘Quadratic Voronoi Diagrams’, wherein every Voronoi cell 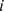 is parameterized by 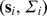 pairs.
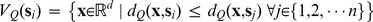
(8)


From Equation(7), it can be observed that 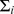 is essentially a weighting factor that non-uniformly weights distances in every Voronoi regions along every dimension. When all the Voronoi regions are equally and uniformly weighted along every axis, 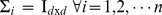 and the resulting diagram for point sites becomes an Euclidean distance based Voronoi diagram.

#### 4.2.1 Estimating The Distance Metric From Sparse Data: Minimum Volume Enclosing Ellipsoid

Now, the problem at hand is to estimate the parameter pair for each cell/quadratic Voronoi regions from the sparse data-points, as obtained from the segmented and tracked slices, that belongs to the boundary of each cell. Given the extreme sparsity of the data, there is no available method that would provide 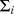
s for each region. We, in this work, propose an alternative way of estimating 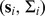 pairs directly from the sparse data-points.

An ellipsoidal surface in 3D is given by the locus of the point 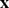 that satisfies
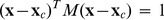
(9)where 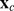 is the center of the ellipsoid and 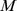 is a positive definite symmetric matrix. For any point 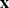 inside the ellipsoid, 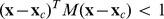 and for every 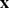 outside it, 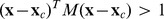.

Now, 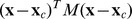 is, in fact, the Mahalanobis distance of the point 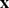 from the center 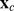 of the ellipsoid. Therefore, if a point 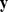 is equidistant for the centers of two ellipsoids 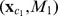 and 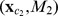 in the Mahalanobis sense, then,
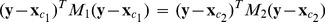
(10)


Now, Equation (10) gives the locus of the points 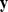, which is exactly the same as that of the points on the bisector two neighbouring quadratic Voronoi regions parameterized by 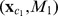 and 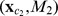. We already have a set of points (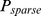)which are sparsely but uniformly z-sampled from the boundaries between neighbouring cells. The tracking algorithm provides us with the exact cell pairs on which every point from this set belongs to. Therefore we can approximately estimate the distance parameters associated with every quadratic Voronoi region individually by fitting an ellipsoid to the sparse data-points belonging to the boundaries of that region. In this paper, we choose fit Minimum Volume Enclosing Ellipsoids (MVEE) to each of 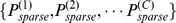 for 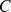 cells individually and obtain approximate estimates of 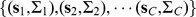. The estimation strategy is described later in this Section and the details of the same can be found in Section 3 of File S1.

As we are estimating the parameters of quadratic distance metric associated with every individual Voronoi cell separately and then using the distance metrics, thus obtained, to tessellate a dense point cloud, we choose to call the resulting Voronoi tessellation as the ‘Adaptive Quadratic Voronoi Tessellation’ (AQVT).

After registration, segmentation and identification of a cell in multiple slices in the 3-D stack, we can obtain 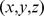 co-ordinates of the set of points on the perimeter of the segmented cell slices. Let this set of points on the 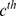 cell be 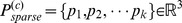. We have to estimate the minimum volume ellipsoid which encloses all these 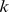 points in 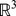 and we denote that with 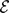. An ellipsoid in its center form is represented by
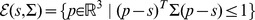
(11)where 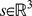 is the center of the ellipsoid 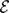 and 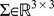. Since all the points in 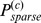 must reside inside 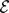, we have
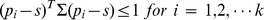
(12)and the volume of this ellipsoid is



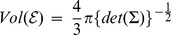
(13)Therefore, the problem of finding the Minimum Volume Enclosing Ellipsoid (MVEE) for the set of points 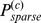 can be posed as
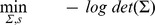


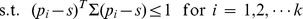


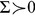
(14)


**Figure 4:**
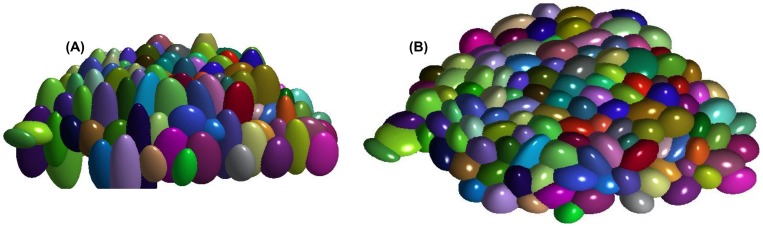
Ellipsoidal representation of the AQVT parameters estimated from the sparse data-points. (A) The Minimum Volume Enclosing Ellipsoids representing the 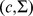 parameter pairs for individual cells are shown in different colors. (B) The same representation viewed from top.

To efficiently solve Problem (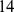) we convert the primal problem into its dual problem since the dual is easier to solve. A detailed analysis on the problem formulation and its solution can be found in [26], [27]. Solving this problem individually for each sparse point set 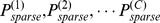, the parameters of the quadratic distance metrics are estimated as 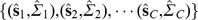. To visually represent these parameters, we have constructed the ellipsoids with each of these parameter pairs and color coded them to represent individual cells in a SAM tissue (Figure 4).

#### 4.2.2 3D Tessellation Based on The Estimated Parameters of AQVT: The Final Cell Shapes

As soon as the parameters of the quadratic distance metrics are estimated from the previous step, the dense point cloud 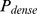 obtained in Section 4.1.3 can be partitioned into different Voronoi regions based on Equation (8), i.e. the dense point cloud belonging to cell 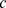 is given as
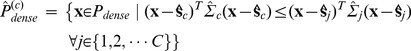
(15)


For visualization purpose of the cell resolution 3D reconstruction results, we fit convex polyhedrons to 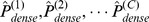 to represent each cell.

#### 4.2.3 Relation Between The AQVT and Deformed Trucncated Ellipsoid Based Tessellation [7]

In a recent work [7], we have shown that the 3D shapes of individual cells in a tightly packed multi-layer tissue can be approximated by deformed truncated ellipsoidal models. In that work, first, the enclosing ellipsoids (MVEE) are fitted to the sparse data-points for each cell, which are then recursively deformed until a certain stopping criterion (see [7]) is satisfied and finally truncated along the overlapping surfaces of those 3D deformed ellipsoids to obtain the final 3D cell shapes.

Following the same notations used before, the estimated enclosing ellipsoids for the sparse point sets 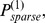 
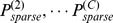
 are given by 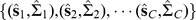. We observed through our experiments that the best 3D reconstruction results are achieved when the estimated ellipsoids are deformed along their axes and the deformation along each axes at each iteration step is proportional to the current length of the axes. Let the factor by which each axis is deformed be 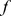 and let there be 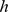 iterations before the algorithm in [7] converges.

As we can express 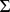 as 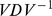 through Eigenvalue decomposition (see [7]) and the deformed 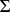 after 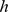 iterations of deformation can be written as
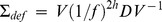
the points on the reconstructed cell boundary between the cells 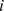 and 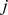, after 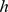 steps of iteration would be given by,
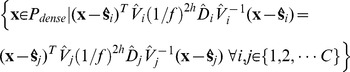
(16)

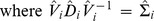


As 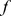 is a scalar, the Expression (16) can be rewritten as,
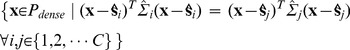
(17)


Equation (17) is exactly same as the set of the boundary points between Voronoi regions 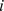 and 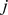 for a Adaptive Quadratic Voronoi Tessellation. Thus, under the deformation condition described above, the method proposed in [7] and AQVT produce the same tessellation result. But, the major advantage of using AQVT over [7] is that, AQVT yields the 3D reconstruction result in a single step, whereas, the deformed truncated ellipsoidal model described in [7] is an iterative process. The number of iterations can be very large depending on the choice of the stopping criterion or the step-size in the deformation stage and hence [7] is, in general, a much slower algorithm. Apart from that, the 3D reconstruction results can vary widely depending on the chosen step-size for deformation in [7] and thus the quality of the reconstruction result is often unpredictable. We have experimentally observed that in [7] the least error in reconstruction is achieved when the deformation step sizes for each cell in the tissue are equal or very close to one another. This condition, as shown above, gives us the distance metric of AQVT. Therefore, AQVT provides a unique solution to the 3D reconstruction and is, in most cases, guaranteed to yield better or the same reconstruction result when compared to that in [7] at a significantly less execution time. It can also be noted that the proposed AQVT does not require any user chosen threshold or parameter to be input into reconstruction framework, making the method less ambiguous and more user friendly than [7].

## Results and Discussion

### 5.1 Pre-processing Results

**Figure 5:**
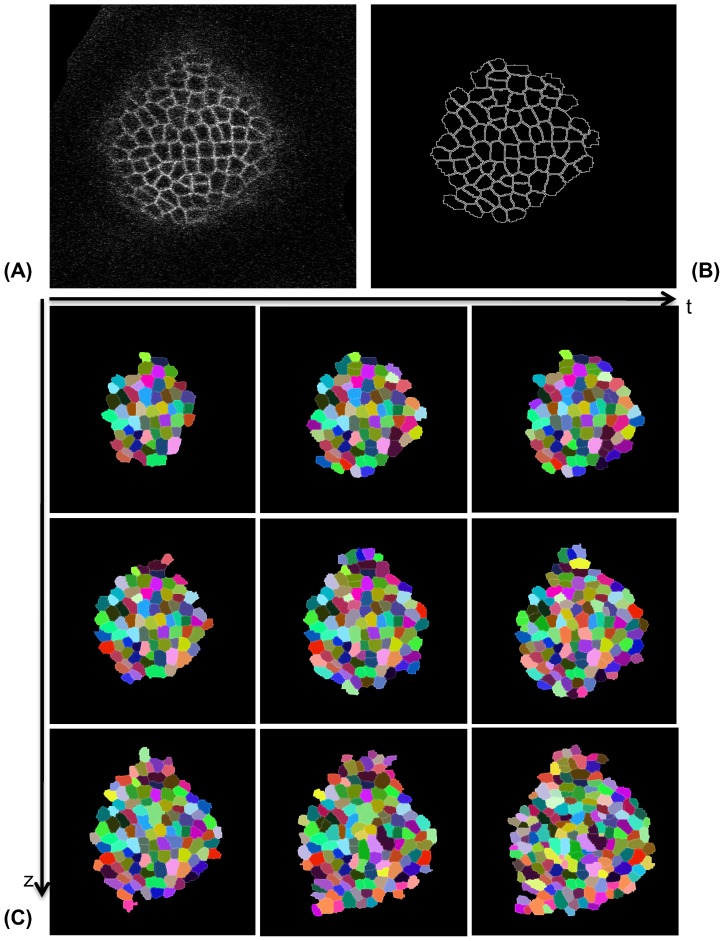
Sample segmentation and spatio-temporal tracking result. (A) Raw confocal image slice, (B) Watershed segmented cell edges from the same image in A, (C) Individual cell slices are tracked in z to find correspondence between slices belonging to the same cells. The cells are color coded in the image to show the correspondences. The cells can also be tracked in time (which is shown using the same color code) that can be useful while reconstructing the same cells in consecutive time points to observe the growth of those cells.

We have tested the proposed 3D Reconstruction framework on a cluster of around two hundred and twenty cells spanning L1 and L2 layers of an Arabidopsis Shoot Apical Meristem. The details of the generation of raw image data using CLSM technique are described in Section 3.1. We used a modified Watershed segmentation [18] to segment individual cell slices and a sample Watershed segmented image slice is shown in Figure 5(B) (the raw image slice is shown in Figure 5(A)). In the next step, we have clustered the slices belonging to each cell using the local graph-matching based cell tracking method described in [4]. An example of the tracking result is shown in Figure 5(C), where the same cells are marked with the same color in three successive slices, in both the spatial and temporal direction. Once the sparse cell slices are clustered together, the sparse set of data points on each cell are extracted and used to estimate the approximate location of the sites for Euclidean distance based Voronoi tessellation or in case of the proposed AQVT based method, the site and weight parameters for the quadratic distance metric for each cell.

### 5.2 3D Reconstruction Results

**Figure 6:**
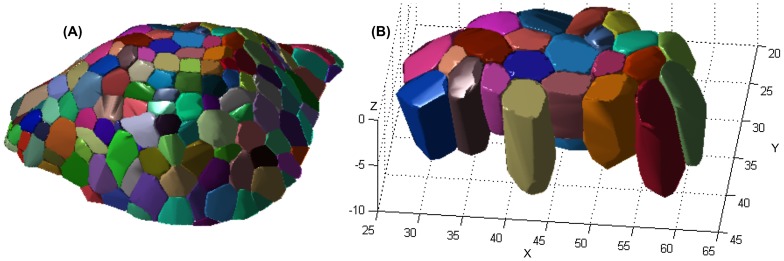
Visualization of the AQVT based 3D reconstruction of SAM cell cluster. (A) Visualization of the 3D reconstructed structure of a cluster of around 220 closely packed cells using convex polyhedron approximations of the densely clustered data-points for each cell, as obtained from the proposed 3D reconstruction scheme, (B) A subset of cells from the same tissue.

Figure 6(A) shows a cell resolution reconstruction of the cell cluster in SAM using AQVT. Note that for 3D visualization purpose of the 3D structure only, we have represented each cell as a convex polyhedron fitted to the dense point cloud clustered to the cells, as obtained from our 3D reconstruction/3D segmentation scheme. For better understanding of the 3D structures of individual cells, we have shown the reconstructed shapes of a smaller cluster of cells in Figure 6(B).

### 5.3 Validation of The Proposed Method

#### 5.3.1 Validation on 3D SAM Data

There is hardly any biological experiment which can directly validate the estimated growth statistics for individual cells in a sparsely sampled multi layered cluster. In fact, the absence of a method to estimate growth statistics directly using non-computational methods in a live-imaging developmental biology framework is the motivation for the proposed work and we needed to design a method for computationally validating our 3D reconstruction technique. Once the 3D reconstruction is achieved, we can computationally re-slice the reconstructed shape along any arbitrary viewing plane by simply collecting the subset of reconstructed 3D point cloud that lies on the plane.

**Figure 7:**
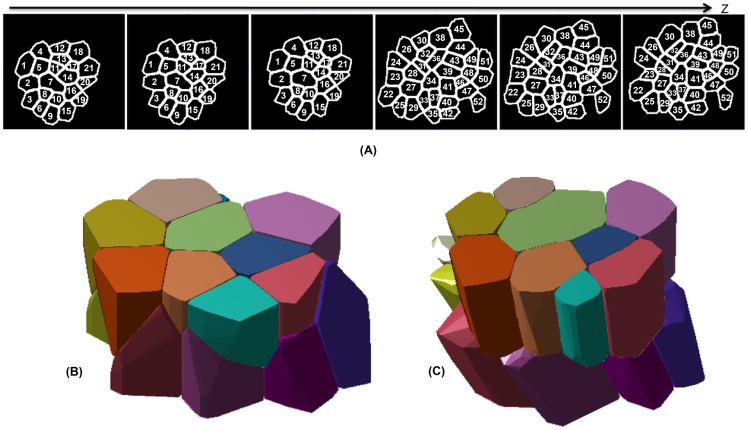
Reconstruction of a cluster of cells using Euclidean distance based Voronoi tessellation and the proposed AQVT for comparison of the 3D reconstruction accuracy. (A) Segmented and tracked cell slices for a cluster of fifty two cells from the L1 and L2 layers of SAM. A dense confocal image stack is subsampled at a z-resolution of 1.35 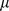
m to mimic the ‘z-sparsity’ observed in a typical Live-Imaging scenario. The slices belonging to the same cell are marked with the same number to show the tracking results. (B) 3D reconstructed structure for a subset of these cells when reconstructed using the Euclidean distance based Voronoi Tessellation. (C) The AQVT based reconstruction result for the same cell cluster.

To show the validation of our proposed method, we have chosen a single time point dataset that is relatively densely sampled along Z (0.225 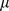
m between successive slices). Then, we resampled this dense stack at a resolution of 1.35 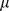
m to generate a sparser subset of slices that mimic the sparsity generally encountered in a live-imaging scenario. The sparsely sampled slices for a cluster of cells spanning two layers (L1 and L2) in the SAM are shown in Figure 7. The aforementioned tracking method [4] is used to obtain correspondences between slices of the same cells. Different slices of the same cells imaged at different depths in Z are shown using the same number in Figure 7(A). Next, we reconstructed the cell cluster first by the standard Voronoi tessellation using the Euclidean distance metric and then using our proposed method (AQVT) with a quadratic distance metric adapted for each of these cells. The reconstruction results for a subset of the cells for each of these methods are shown in Figures 7(B) and 7(C) respectively for a direct comparison. It can be observed that not only our proposed method very accurately reconstructed the cell shapes but also it has captured the multi-layer architecture of these SAM cells more closely in comparison to its Voronoi counterpart with the Euclidean distance metric.

To better investigate the accuracy of the reconstruction and to show the clear advantage of using the proposed method of reconstruction quantitatively, we measure distances between the 2D cross sections of the 3D reconstruction results for each cell to the Watershed segmented cell boundaries. By choosing the reslicing plane as 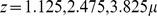
m (different slices than those used for reconstruction) from top of the stack for the L1 layer cells and 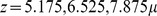
m for the L2 layer cells, we can computationally regenerate the cell walls for these imaging planes along Z. The shapes of cells in the reconstructed slices can be compared against their counterparts in the 2D segmented images and the distance between the shapes would represent the reconstruction error. There are several different distance metrics that can be used to compute the dissimilarity between two shapes such as the Procrustes distance, Hausdorff distance etc., each one having its own advantages. We have chosen to use the Modified Hausdorff Distance (MHD), one of the more popular distance measures in the Hausdorff distance family, to evaluate our reconstruction method. The advantages of MHD over other distance measures for object shape matching is described in details in [28]. It can be noted that the error, thus computed, is analogous to the reprojection error that is widely used in the 3D reconstruction community to quantify the accuracy of reconstruction.

**Figure 8:**
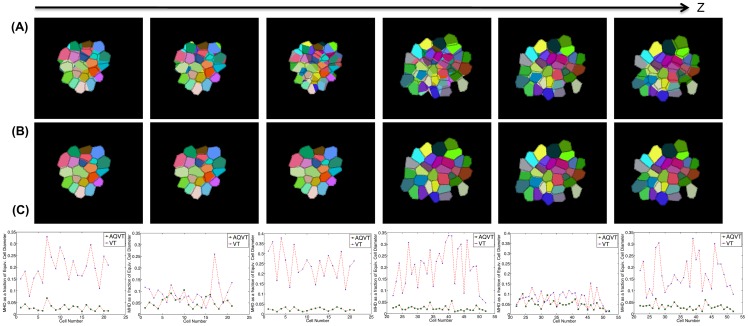
Comparison of the 3D reconstruction accuracy for the proposed AQVT based reconstruction against Euclidean distance based Voronoi tessellation. (A) The cells shown in Figure 7(A) are reconstructed using the Euclidean distance based Voronoi tessellation and the computationally re-sliced cells are compared against the ground truth. (B) The same cells are reconstructed using the adaptive quadratic distance based Voronoi tessellation and then computationally re-sliced along various depths in z at which we also have the ground truth (in terms of the 2D segmentation results of the cell slices), but were not used in generating the reconstruction results. The computationally obtained cell slices are shown in different colors for different cells and they are superimposed by the ground truth segmentation results. (C) The error in reconstruction (similar to the reprojection error) is computed as the Modified Hausdorff Distance (MHD) between the computationally generated cell slices and the segmentation results on the ground truth images of the same cells. The MHD, computed for each of the 52 cells at different depths in the Z-stack are plotted for both the methods to compare the methods against each other. It can be clearly observed from the plots that the reconstruction error is much larger for the Euclidean distance based Voronoi tessellation (VT) than for AQVT, especially at the terminal (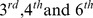) slices, between consecutive layers of cells.

In Figure 8(A), we have shown the computationally resliced cell slices (color coded to represent the same cells at multiple slices) at various depths for Euclidean distance based Voronoi tessellation and Figure 8(B) shows the 2D cross sections for the same cells as obtained by reslicing the 3D cell shapes in the proposed AQVT based reconstruction method. For both the image sets, the computationally obtained cross sections of each cell are superimposed by the Watershed segmentation results for the same cell slices (the ground truth). The MHD between the original and computationally generated cell slices are computed and for each of the cells in 6 different slices, this error is shown in Figure 8(C).

It can be observed that each of the graphs in Figure 8(C) comprises of the reconstruction errors for individual cell slices for both the methods of the reconstruction. For the slices closer to the center of the cells, both the methods show similar and acceptably small reprojection error. However, in case of the terminal slices for each cell along Z, the proposed adaptive quadratic distance based reconstruction method shows a far better reconstruction accuracy (as evident in the 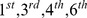 graphs in Figure 8(C)). This improvement of result is more prominent for cells that have non-uniform shapes and growth such as elongation along any one of the axis. We further validate this observation by performing a similar experiment on a cluster of cells of various sizes and dimensions in a sample root meristem longitudinal cross sectional slice image. This experiment and the results are elaborated in the next subsection.

**Figure 9:**
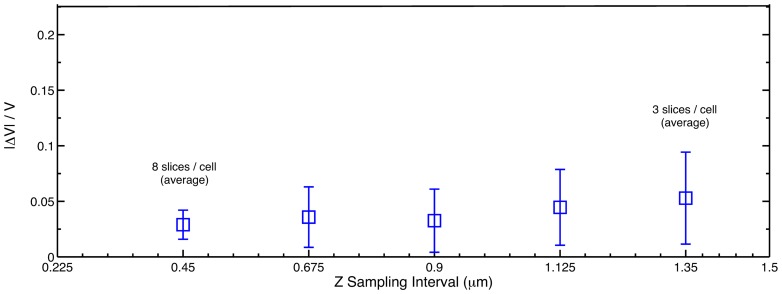
Errors in AQVT estimated cell volumes from their respective ground truth volumes at various levels of sparsity. A cluster of cells from a 3D confocal image stack with z resolution of 0.225 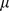
m is resampled to generate stacks of 5 different levels of sparsity. Each of these resampled stacks is 3D reconstructed using the proposed AQVT and volumes of each of the cells in the cluster are computed. The means and standard deviations of absolute errors in volumes (expressed as a ratio to the ground truth volumes) of all the cells for each sparser stacks are plotted. The average error slowly increases with increased sparsity but is less than 5.3% with a standard deviation of 4% even at 1.35 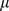
m/slice (i.e. 3 slices/cell on an average).

We have also evaluated the accuracy of our method by studying how much do the estimated volumes of the cells differ from ground truth for various levels of sparsity in the z-sampling. For the dense data described before (0.225 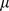
m between slices along z), we first estimate the ground truth cell volumes of a cluster of cells by counting the total number of superpixels per cell and multiplying that with the superpixel size (0.2×0.2×0.225 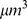). Then, we gradually resample the dense stack at 5 successive z resolutions (viz. 0.45, 0.675, 0.9, 1.125, 1.35 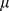
m) and for each of these resampled stacks, we reconstruct the same cells using the proposed AQVT and estimate the cell volumes. It can be noted that with each of these respective resampling, the resultant 3D stack becomes more and more sparse. For example, at 0.45 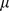
m z resolution of sampling, the average number of slices per cell is 7 or 8, whereas, for 1.35 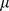
m, the same cells are captured in an average of 3 slices. The errors in estimation of individual cell volumes at various sparsity levels are shown in Figure 9. We have plotted the means and standard deviations of the absolute errors expressed as a ratio to the ground truth cell volumes. It can be observed that at the densest resampling (0.45 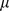
m), the average estimation error is as low as 3% with a standard deviation of 1.3%. The estimation error slowly increases with increased sparsity in the stack and at a sparsity of about 3 slices/cell, the average estimation error is 5.3%, with standard deviation of around 4%. We have repeated the same experiment for Voronoi tessellation with Euclidean distance metric and the average error is much higher at around 30% (see Figure S1).

#### 5.3.2 Validation On 2D Root Meristem Data

**Figure 10:**
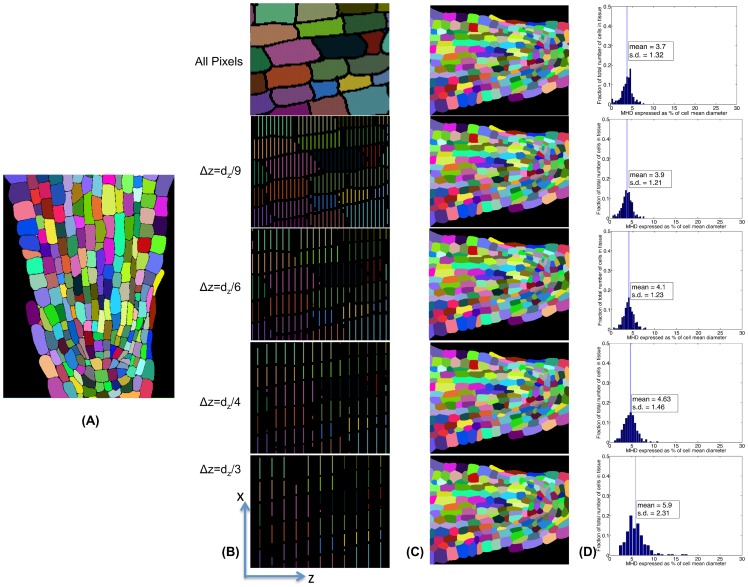
Validation of AQVT on 2D root apex longitudinal cross section data. (A) Ground truth segmentation of a sample cross sectional slice of root apical meristem tissue (the source images for this tissue can be found in [29]). (B) The zoomed in tissue after segmentation (B-top) and sparser point clouds per cell (in the 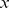-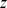 plane) after resampling the tissue at various 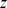-resolutions (zoomed in for clarity). (C) The cells (color-coded) are reconstructed using the proposed AQVT with the resampled point clouds as shown in B to present the change in reconstruction quality with increased sparsity in the sampled point clouds for both the larger and elongated cells towards the outer and upper part and the smaller cells towards the lower central part of the tissue. (D) Quantitative measure of reconstruction errors: the difference between actual and reconstructed cell shapes are computed using modified Hausdorff distance (MHD) and the histograms of MHDs for all the cells at every level of sparsity is plotted.

For further evaluating the performance of the proposed AQVT on tightly packed cells of heterogeneous sizes and shapes, we perform similar experiments on a 2D image of root meristem longitudinal cross section with 226 cells. The ground truth segmentation of the tissue is shown in Figure 10(A), the raw images for which can be seen in Figure 5 of [29]. From Figure 10(A), it can be seen that a large number of cells in the tissue slice (shown as 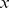-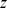 plane in Figure 10(B)) is more elongated along the longitudinal direction (shown as 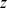-axis in Figure 10(B)), whereas the other cells have similar 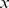 and 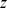 diameters. Again, the cells towards the the periphery are much larger than the cells in the lower central region of the tissue. To mimic the scenario of sparse sampling along one dimension, we artificially sample the segmented cells along 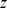 (longitudinal) axis (see Figure 10(B)) to generate point clouds for each cell at various sparsity (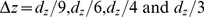, where 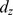 is the average cell diameter along 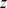 axis). We assume sparse clustering of sampled points per cell (analogous to spatial tracking in 3D) is given to us as the contribution of the present work is dense reconstruction of cells from sparse point clouds, which lies in the post segmentation and tracking stage of the reconstruction pipeline. For each of the resampled point clouds, we use AQVT (in 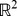) to reconstruct the cells (Figure 10(C)). We compute modified Hausdorff distances from every reconstructed cell shape to the ground truth segmentation for quantitative evaluation of the reconstruction accuracy (Figure 10(D)). We can observe that the mean error in the reconstructed cell shapes is less than 6% of average cell longitudinal diameter and the maximum reconstruction error for most of the cells in the tissue is less that 10% for all sparsities upto 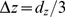 (average 3 slices or lines/cell).

## Conclusion and Future Work

In this paper, we have presented a method of reconstructing densely packed cluster of cells using a very sparsely z-sampled confocal live imaging dataset. We have provided a mathematically rigorous framework built on top of basic geometric tessellation concepts. We have first shown how the cell shapes can be approximated by the Voronoi tessellation based on Euclidean distance measure. Then, we proposed a quadratic distance metric based Voronoi tessellation framework to capture the asymmetry of the cell sizes and growth along their different axes. We described how the proposed tessellation can take care of the asymmetry by providing weights on the distance metric along each axis for each cell and how these weights as well as the location of the sites can be approximately estimated from the sparse image data for individual cells by fitting enclosing ellipsoids to the segmented sparse image slices. We have validated our method by showing that the reconstruction error (both in reconstructed cell shapes and estimated cell volumes) for the cells is sufficiently low and have provided a direct comparison of the reconstruction error for the proposed method against the popular Euclidean distance based Voronoi tessellation approach. As an application of the proposed method, we have shown some preliminary results on the estimation of growth curves for a few SAM cells in Figure S2. Future work on this would include the integration of this 3D reconstruction method with the spatio-temporal cell tracking method to form a complete 4D image analysis pipeline. This pipeline could be used to generate various cell division and cell growth statistics in a fully automated, high-throughput manner. The statistics collected from such a pipeline could be extremely useful in building a dynamical model to quantitatively analyse the spatio-temporal correlation in cell division and cell growth in a complex multi-layered tissue.

## Supporting Information

Figure S1
**Errors in estimated cell volumes using Euclidean distance based Voronoi tessellation from their respective ground truth volumes at various levels of sparsity.** A cluster of cells from a 3D confocal image stack with z resolution of 0.225 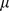
m is resampled to generate stacks of 5 different levels of sparsity. Each of these resampled stacks is 3D reconstructed using the Euclidean distance based VT and volumes of each of the cells in the cluster is computed. The means and standard deviations of absolute errors in volumes (expressed as a ratio to the ground truth volumes) of all the cells for each sparser stacks are plotted. The average error is more or less similar at all sparsity levels and the average error is around 30% with a large standard deviation of more than 25%).(TIFF)

Figure S2 **Cell Growth Curves.** Growth curves for five sample cells after the removal of occasional outliers.
(TIFF)

File S1 **Supporting Information Text File.** Contains an analysis of cell volume estimation errors in Euclidean distance based Voronoi tessellation, sample results on cell growth statistics and a detailed solution strategy for the estimation of MVEE parameters.
(PDF)

File S2 **Codes and Demo.** Contains a MATLAB implementation of AQVT as well as a working demo of the codes on a sample 3D confocal stack of Arabidopsis SAM.
(ZIP)
