# Supplementary figures and images for: Adaptive Geometric Tessellation for 3D Reconstruction of Anisotropically Developing Cells in Multilayer Tissues from Sparse Volumetric Microscopy Images
Source: PLoS One. 2013 Aug 5;8(8):e67202. doi: 10.1371/journal.pone.0067202 (PMC3734189; doi:10.1371/journal.pone.0067202)

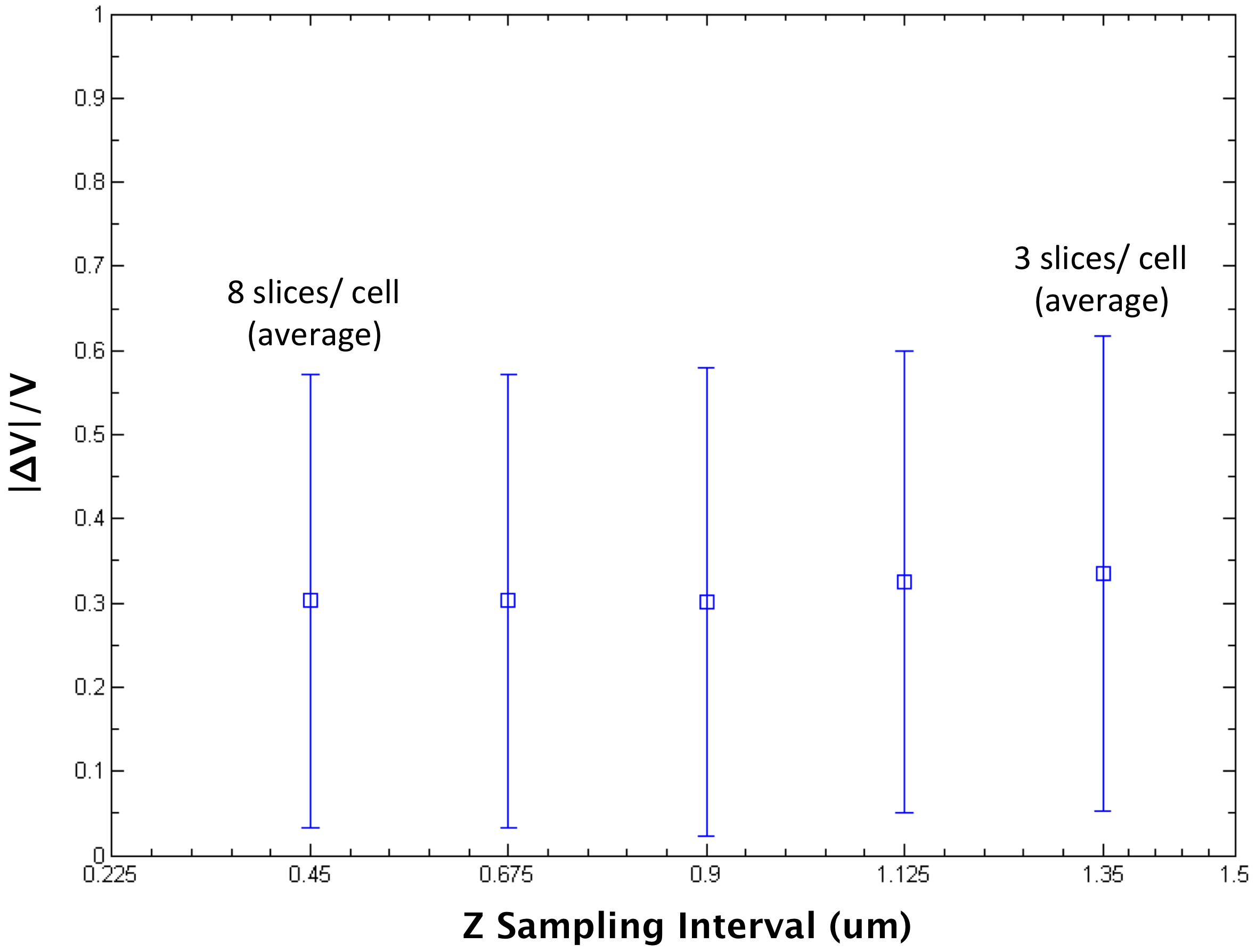

Supplement: Figure S1 — Errors in estimated cell volumes using Euclidean distance based Voronoi tessellation from their respective ground truth volumes at various levels of sparsity. A cluster of cells from a 3D confocal image stack with z resolution of 0.225 m is resampled to generate stacks of 5 different levels of sparsity. Each of these resampled stacks is 3D reconstructed using the Euclidean distance based VT and volumes of each of the cells in the cluster is computed. The means and standard deviations of absolute errors in volumes (expressed as a ratio to the ground truth volumes) of all the cells for each sparser stacks are plotted. The average error is more or less similar at all sparsity levels and the average error is around 30% with a large standard deviation of more than 25%). (TIFF) [file pone.0067202.s001.tiff]

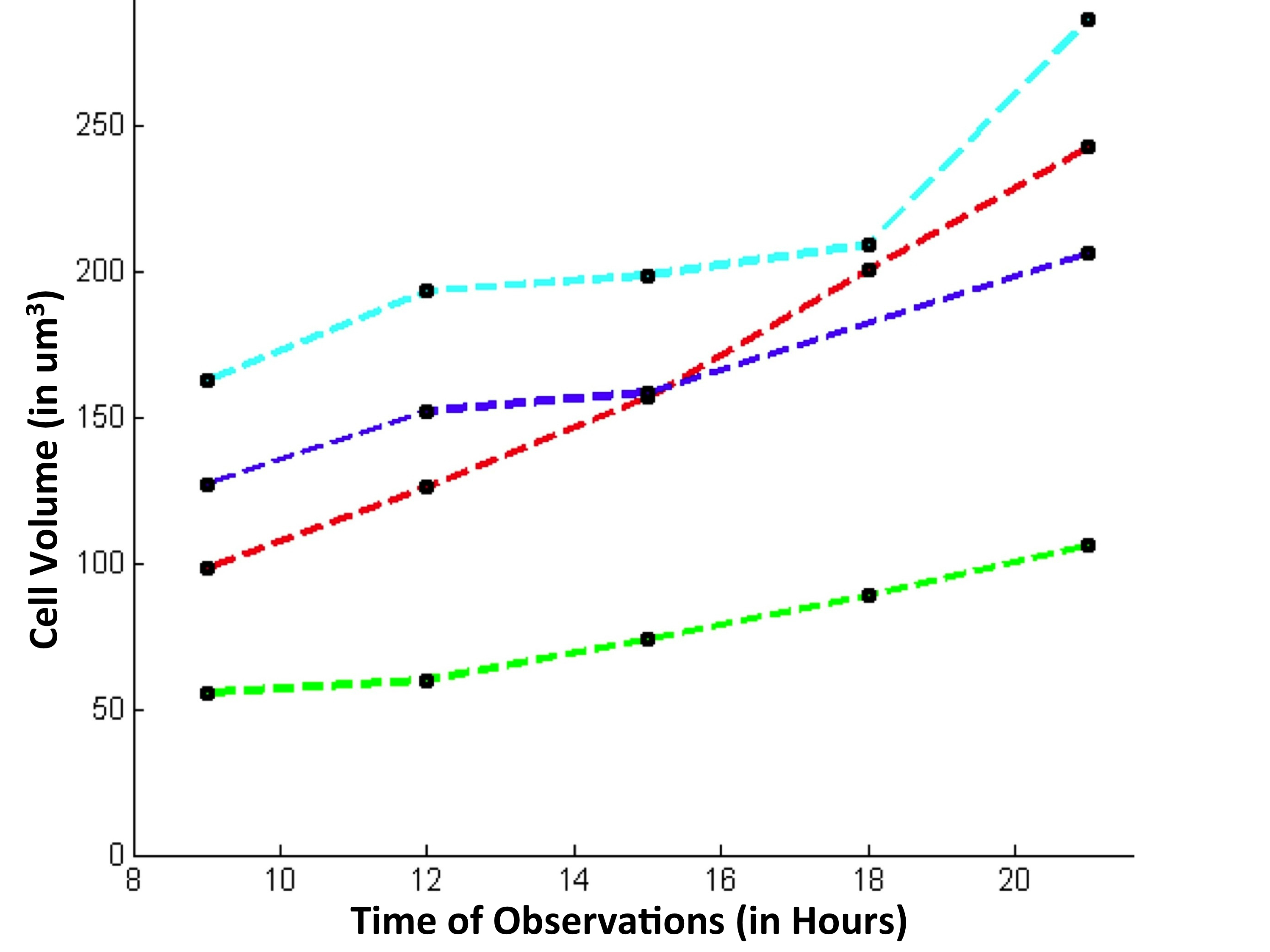

Supplement: Figure S2 — Cell Growth Curves. Growth curves for five sample cells after the removal of occasional outliers. (TIFF) [file pone.0067202.s002.tiff]
