# Supplementary material for: Adaptive Geometric Tessellation for 3D Reconstruction of Anisotropically Developing Cells in Multilayer Tissues from Sparse Volumetric Microscopy Images
Source: PLoS One. 2013 Aug 5;8(8):e67202. doi: 10.1371/journal.pone.0067202 (PMC3734189; doi:10.1371/journal.pone.0067202)
Supplement: File S1 — Supporting Information Text File. Contains an analysis of cell volume estimation errors in Euclidean distance based Voronoi tessellation, sample results on cell growth statistics and a detailed solution strategy for the estimation of MVEE parameters. (PDF) [file pone.0067202.s003.pdf]

Supplementary File S1

1 Absolute Errors in Volume Estimation For Euclidean Distance Based Voronoi Tessellation

We evaluate the accuracy of the cell volume estimates obtained by using Euclidean distance based Voronoi tessellation on sparsely z-sampled cell slices of Arabidopsis SAM through an experiment exactly similar to that described in the Section 5.3.1. From a denser stack with z-resolution of 0.225 μm, we resample slices at various levels of sparsity (at z-resolutions of 0.45, 0.675, 0.9, 1.125, 1.35 μm) and then reconstruct the cells using the standard Voronoi tessellation technique (Section 4.1.1). The estimated cell volumes at each sparsity level are compared to their respective ground truth and errors are computed as the absolute difference in estimated volume expressed as a ratio to the ground truth volumes. Finally, means and standard deviations of these errors for all the cells in each resampled stack are plotted in Figure S1.

2 Cell Growth Statistics

Useful growth statistics such as the cell growth curves can be obtained as a direct application of the 3D reconstruction method. We looked at the evolution of the individual cell volumes in time by reconstructing the same cell clusters at successive time points and some results are shown in Figure S2. If a sufficiently dense point cloud is partitioned into individual cells in the 3D reconstruction framework, the volume of the complex cell shapes can be very accurately estimated by summing the volumes of 3D cubic voxels around each point belonging to that cell. The growth curves for all the cells show similar growth rates on an average. This is biologically valid as all the cells are chosen from a close neighbourhood in SAM. Note that the accuracy in the computation of cell growth statistics is affected by the spatial and temporal cell tracking as an error in obtaining correspondences between cell slices is directly propagated into the 3D reconstruction cum cell volume estimation framework. The fact that a cell's volume should increase monotonically could be utilized in improving the tracker's performance. However, the improvements of the tracking methods should be dealt separately in the cell tracking literature and is beyond the scope of this paper. Other cell size statistics, such as the distributions of the cell volumes in clonally different regions across the tissue (such as central zone and peripheral zone in the SAM) can also be estimated.

3 Detailed Estimation of MVEE Parameters

Here we provide the detailed solution [1] to the optimization problem in (14) for estimation of the MVEE parameters. We define a 'lifting' from $\mathcal{P} \in \mathbb{R}^3$ to $\mathbb{R}^4$ via

$$\mathcal{P}^{'} \;=\; \{\pm q_1,\ \pm q_2,\ \cdots\ \pm q_n\}$$

where $q_i = [p_i, 1]^T,\ i = 1, 2, \cdots n$. Now, we define a hyperplane $\mathcal{H} = \big\{(p,\ p_4) \in \mathbb{R}^4 : p_4 = 1\big\}$ such that

$$MVEE(\mathcal{P}) \;=\; MVEE(\mathcal{P}^{'}) \;\cap\; \mathcal{H} \tag{1}$$

MVEE($\mathcal{P}^{'}$) is centered at origin and this helps us in changing the problem in (14) in the main manuscript as a convex optimization problem for MVEE($\mathcal{P}^{'}$). Once this problem is solved we can find out MVEE($\mathcal{P}$) using Relation (1) above. Now, the lifted problem is

$$\begin{aligned} \min_{A} \quad & -log\ det(A) \\ \text{s.t.} \quad & {q_i}^T A q_i \leq 1 \text{ for } i = 1, 2, \cdots n \\ & A \text{ is a } 4{\times}4 \text{ positive definite symmetric matrix} \end{aligned} \tag{2}$$

The Lagrangian dual problem of the above:

$$\begin{aligned} \max_{u} \quad & \textit{log det } F(u) \\ \text{s.t.} \quad & \underline{1}^T u = 1 \\ & u \geq 0 \end{aligned} \tag{3}$$

where $u \in \mathbb{R}^n$ is the decision variable and 'F' is a linear operator such that

$$F(u) = \sum_{i=1}^{n} u_i q_i {q_i}^T \tag{4}$$

Problem (3) is a concave optimization problem which can be solved by an ascent method. Based on the work by L.G. Khachiyan in [2], a conditional gradient ascent method [3] can be employed to solve this optimization problem.

Now, $\hat{u}$ is an optimal solution to the optimization problem along with the dual solutions $\hat{s} \in \mathbb{R}^n$ and $\hat{\lambda} \in \mathbb{R}$ if and only if the following conditions are satisfied [3]

$${q_i}^T F(\hat{u})^{-1} q_i + \hat{s_i} = \hat{\lambda} \text{ for } i = 1, 2, \cdots n \tag{5}$$

$$\underline{1}^T \hat{u} = 1 \tag{6}$$

and

$$\hat{u_i}\hat{s_i} = 0 \text{ for } i = 1, 2, \cdots n \tag{7}$$

where $\hat{u},\ \hat{s} > 0$. Then, multiplying both sides of Equation (5) by $\hat{u_i}$ and summing up over all i gives

$$\sum_{i=1}^{n} \hat{u_i} {q_i}^T F(\hat{u})^{-1} q_i = \text{Trace}\left[F(\hat{u})^{-1}\left(\sum_{i=1}^{n} \hat{u_i} q_i {q_i}^T\right)\right] = \text{Trace(I)}$$

Now I is a 4×4 matrix and hence Trace(I)=4. This implies $\hat{\lambda} = 4$. Consequently,

$$A = \frac{1}{4} F(\hat{u})^{-1} \tag{8}$$

Combining the results, the expression of MVEE:

$$MVEE(\mathcal{P}) = \{p \in \mathbb{R}^3 \mid \frac{1}{4} \begin{bmatrix} p^T & 1 \end{bmatrix} F(\hat{u})^{-1} \begin{bmatrix} p \\ 1 \end{bmatrix} \leq 1\} \tag{9}$$

Now, let $\mathrm{P} \in \mathbb{R}^{3 \times n}$ such that the i'th column of P is p_i. Then, $F(\hat{u})$ from Equation (4) can be alternatively expressed in a matrix form in terms of P -

$$F(\hat{u}) = \begin{bmatrix} \mathrm{P}\hat{\mathrm{U}}\mathrm{P}^T & \mathrm{P}\hat{u} \\ (\mathrm{P}\hat{u})^T & 1 \end{bmatrix}$$

where, $\mathrm{U} = \text{diagonal}(u) \in \mathbb{R}^{n \times n}$. Upon inversion,

$$F(\hat{u})^{-1} = \begin{bmatrix} \mathrm{I} & 0 \\ (-\mathrm{P}\hat{u})^T & 1 \end{bmatrix} \begin{bmatrix} (\mathrm{P}\hat{\mathrm{U}}\mathrm{P}^T - \mathrm{P}\hat{u}(\mathrm{P}\hat{u})^T)^{-1} & 0 \\ 0 & 1 \end{bmatrix} \begin{bmatrix} \mathrm{I} & -\mathrm{P}\hat{u} \\ 0 & 1 \end{bmatrix}$$

Plugging the value of $F(\hat{u})^{-1}$ into (9), we get $MVEE(\mathcal{P})$ as

$$\{p \in \mathbb{R}^3 \mid (p - \mathrm{P}\hat{u})^T \frac{1}{3} (\mathrm{P}\hat{\mathrm{U}}\mathrm{P}^T - \mathrm{P}\hat{u}(\mathrm{P}\hat{u})^T)^{-1} (p - \mathrm{P}\hat{u}) \leq 1\} \tag{10}$$

Hence, the estimated parameters of the $MVEE(\mathcal{P})$ are

$$\hat{\Sigma} = \frac{1}{3} (\mathrm{P}\hat{\mathrm{U}}\mathrm{P}^T - \mathrm{P}\hat{u}(\mathrm{P}\hat{u})^T)^{-1} \text{ and } \hat{c} = \mathrm{P}\hat{u} \tag{11}$$

References

1. Moshtagh N Minimum volume enclosing ellipsoids .
2. Khachiyan LG (1996) Rounding of polytopes in the real number model of computation. Math Oper Res : 307–320.
3. Kumar P, Yildirim EA (2005) Minimum-volume enclosing ellipsoids and core sets. Journal of Optimization Theory and Applications 126: 1–21.
